# RETRACTION: Association Between Stroke And Venous Leg Ulcers: A Mendelian Randomization Study

**DOI:** 10.1111/iwj.70656

**Published:** 2025-04-23

## Abstract

**RETRACTION:** XeR. , HanZ. , LiuG. , and ChenL. , “Association Between Stroke And Venous Leg Ulcers: A Mendelian Randomization Study,” International Wound Journal 21, no. 3 (2024): e14785, 10.1111/iwj.14785.38414343 PMC10899862

The above article, published online on 27 February 2024, in Wiley Online Library (http://onlinelibrary.wiley.com/), has been retracted by agreement between the journal Editor in Chief, Professor Keith Harding; and John Wiley & Sons Ltd. Following an investigation by the publisher, all parties have concluded that this article was accepted solely on the basis of a compromised peer review process. The editors have therefore decided to retract the article. The authors did not respond to our notice regarding the retraction.

**RETRACTION:** R. Xe , Z. Han , G. Liu , and L. Chen , “Association Between Stroke And Venous Leg Ulcers: A Mendelian Randomization Study,” International Wound Journal 21, no. 3 (2024): e14785, 10.1111/iwj.14785.38414343 PMC10899862

The above article, published online on 27 February 2024, in Wiley Online Library (http://onlinelibrary.wiley.com/), has been retracted by agreement between the journal Editor in Chief, Professor Keith Harding; and John Wiley & Sons Ltd. Following an investigation by the publisher, all parties have concluded that this article was accepted solely on the basis of a compromised peer review process. The editors have therefore decided to retract the article. The authors did not respond to our notice regarding the retraction.

